# The Effect of 17β-Estradiol on Cutaneous Wound Healing in Protein-Malnourished Ovariectomized Female Mouse Model

**DOI:** 10.1371/journal.pone.0115564

**Published:** 2014-12-17

**Authors:** Kanae Mukai, Emi Komatsu, Yukari Nakajima, Tamae Urai, Nasruddin, Junko Sugama, Toshio Nakatani

**Affiliations:** 1 Department of Clinical Nursing, Graduate Course of Nursing Science, Division of Health Sciences, Graduate School of Medical Sciences, Kanazawa University, Kanazawa, Japan; 2 Faculty of Health Sciences, Institute of Medical, Pharmaceutical and Health Sciences, Kanazawa University, Kanazawa, Japan; Ohio State University, United States of America

## Abstract

Cutaneous wound healing is delayed by protein malnutrition (PM). On the other hand, estrogen promotes cutaneous wound healing by its anti-inflammatory and cell proliferation effects. Therefore, we hypothesized that estrogen administration in protein-malnourished ovariectomized (OVX) female mice might improve the inflammatory response and promote cutaneous wound healing as well as normal nutrition. To test this hypothesis, we used full-thickness excisional wounds in Control SHAM, PM SHAM, PM OVX and PM OVX+17β-estradiol mice. The Control diet included 200 g/kg protein and the PM diet included 30 g/kg protein. The ratio of wound area in the Control SHAM group was significantly smaller than those in the three PM groups. In addition, microscopic findings also showed that the ratio of collagen fibers, the ratio of myofibroblasts and the number of new blood vessels in the Control SHAM group were significantly greater than those in the three PM groups. However, the number of Ym1-positive cells as an anti-inflammatory M2-like macrophage marker in the PM OVX+17β-estradiol group was significantly higher than those in the other three groups. These results indicate that the appearance of anti-inflammatory M2-like macrophages was promoted by estrogen administration; however, it could not promote cutaneous wound healing upon a low-protein diet. Therefore, it may be confirmed that nutrition is more important for promoting cutaneous wound healing than estrogen administration.

## Introduction

Cutaneous wound healing is a complex tightly orchestrated response to injury, carefully regulated at temporal and spatial levels [1]. There are three major stages: inflammation, proliferation and remodeling. In particular, the inflammation phase is regarded as a critical period of cutaneous wound healing, essential for clearing away contaminating bacteria and creating an environment conducive to subsequent events such as tissue repair and regeneration [2]–[4].

However, various factors are related to cutaneous wound healing [5]. Among these, malnutrition is a major health issue affecting people in developed countries: more than 50% of the elderly in hospitals and institutions were found to be malnourished or at risk of malnutrition [6], [7]. Protein plays a major role throughout the cutaneous wound healing process. Tsuda et al. reported that wound area was larger throughout wound healing in mice fed a protein-free (0 g/kg) rather than a control diet (200 g/kg) [8]. Lim et al. reported that wound size was larger throughout the wound healing period and inflammatory response was delayed with decreased expression of TNF-α and IL-1β and decreased neutrophil infiltration in mice fed a protein malnutrition (PM) diet (5 g/kg) compared with those of mice fed a control diet (150 g/kg) [9]. Otranto et al. reported that inflammatory cells were present at a higher level and wound contraction, collagen deposition and neovascularization were impaired in a protein-restricted group (0 g/kg) compared with those in a control group (230 g/kg) [10]. Moreover, Otranto et al. also reported that inflammatory cells, collagen deposition and neovascularization were disturbed in a slight-protein-restriction group (120 g/kg) compared with those in a control group [10]. These lines of research indicate that the entire process of cutaneous wound healing is delayed under malnutrition due to protein-free or low-protein conditions, and cutaneous wound healing is disturbed by slight protein malnutrition.

On the other hand, female sex hormones also affect cutaneous wound healing. In menopausal women, cutaneous wound healing was shown to be delayed and inflammatory response was prolonged by a dramatic reduction of estrogen, which induces an increase of inflammatory cells; however, it was reversed by the topical replacement of estrogen, which induces a decrease of inflammatory cells [11]. In addition, in young ovariectomized (OVX) female rodents under normal nutrition, cutaneous wound healing was delayed compared with that in SHAM mice by increasing inflammatory cells and TNF-α expression [12]–[18]; however, estrogen administration reversed this delay by reducing neutrophils, macrophages and the expression of TNF-α [13]–[18]. Moreover, estrogen was shown to promote cutaneous wound healing by increasing Ym1-positive cells [16], which are thought of as anti-inflammatory M2-like macrophages, being involved in tissue repair rather than classical tissue inflammation by producing anti-inflammatory cytokines, growth factors and ECM [19]–[22] and the expression of TGF-β1 [12], [17], and promoting collagen deposition [12], [14]. In addition, from our previous research, it was also reported that estrogen administration promoted cutaneous wound healing in OVX mice under normal nutrition by reducing neutrophils and macrophages [23], [24] and promoting collagen deposition [23], [24] and wound contraction [24] at 24 weeks and 40 weeks.

Therefore, we thought that the anti-inflammatory effect and cell proliferation action due to estrogen would be shown upon low-protein malnutrition, such as having a small amount of endogenous protein. Specifically, we hypothesized that estrogen administration in low-protein-malnourished OVX female mice might improve the inflammatory response and promote cutaneous wound healing as well as in normal nutrition. To test this hypothesis, we used full-thickness excisional wounds in OVX mice fed a 3% protein diet (30 g/kg) with estrogen administration to examine the wound area, numbers of neutrophils, macrophages and Ym1-positive cells, expression of plasma TNF-α, expression of plasma CD163 as an marker of anti-inflammatory M2-like macrophages [25], [26], collagen deposition, angiogenesis and wound contraction. The aim of this study was to evaluate whether inflammatory response is improved and cutaneous wound healing is promoted by 17β-estradiol administration in OVX female mice fed a 3% protein diet.

## Materials and Methods

### Animals and diets

Ninety C57BL/6 female mice aged 7 weeks (Sankyo Lab Service Co., Tokyo, Japan) were used in the experiments. They were caged individually in an air-conditioned room at 25.0±2.0°C with light from 08:45 to 20:45 hours, and water and chow were given freely. All animal experiments conducted in this study were reviewed and approved by Kanazawa University Animal Experiment Committee, and carried out in accordance with the Guidelines for the Care and Use of Laboratory Animals of Kanazawa University, Japan (AP-132740). We used three different diets, which were CRF-1 rodent diet (normal diet, 22.6% protein), AIN-93G purified rodent diet (control diet, 20% protein) and 3% modified AIN-93G purified rodent diet (protein malnutrition (PM) diet, 3% protein) (Oriental Yeast Co. Ltd., Tokyo, Japan). Purified rodent diets of AIN-93G and 3% modified AIN-93G were made by Oriental Yeast Co. Ltd. The diet compositions are shown in Tables 1 and 2.

**Table 1:** Diet composition.

	CRF-1	AIN-93G	3% modified AIN-93G
	(Normal diet)	(Control diet)	(PM diet)
Protein (%)	22.6	20.0	3.0
Kcal (per 100 g)	356.0	377.2	377.2

Oriental Yeast Co. Ltd.

**Table 2:** The ingredients of the AIN-93G and 3% modified AIN-93G rodent diets.

	AIN-93G	3% modified AIN-93G
	(Control diet)	(PM diet)
Casein (%)	20.0	3.0
L-Cystine (%)	0.3	0
Cornstarch (%)	39.7486	49.2486
Chemical cornstarch A (%)	13.2	21.0
Sucrose (%)	10.0	10.0
Soya bean oil (%)	7.0	7.0
Cellulose powder (%)	5.0	5.0
AIN-93G containing minerals (%)	3.5	3.5
AIN-93G containing vitamins (%)	1.0	1.0
Choline bitartrate (%)	0.25	0.25
T-butylhydroquinone (%)	0.0014	0.0014

Oriental Yeast Co. Ltd.

**Figure 1:**
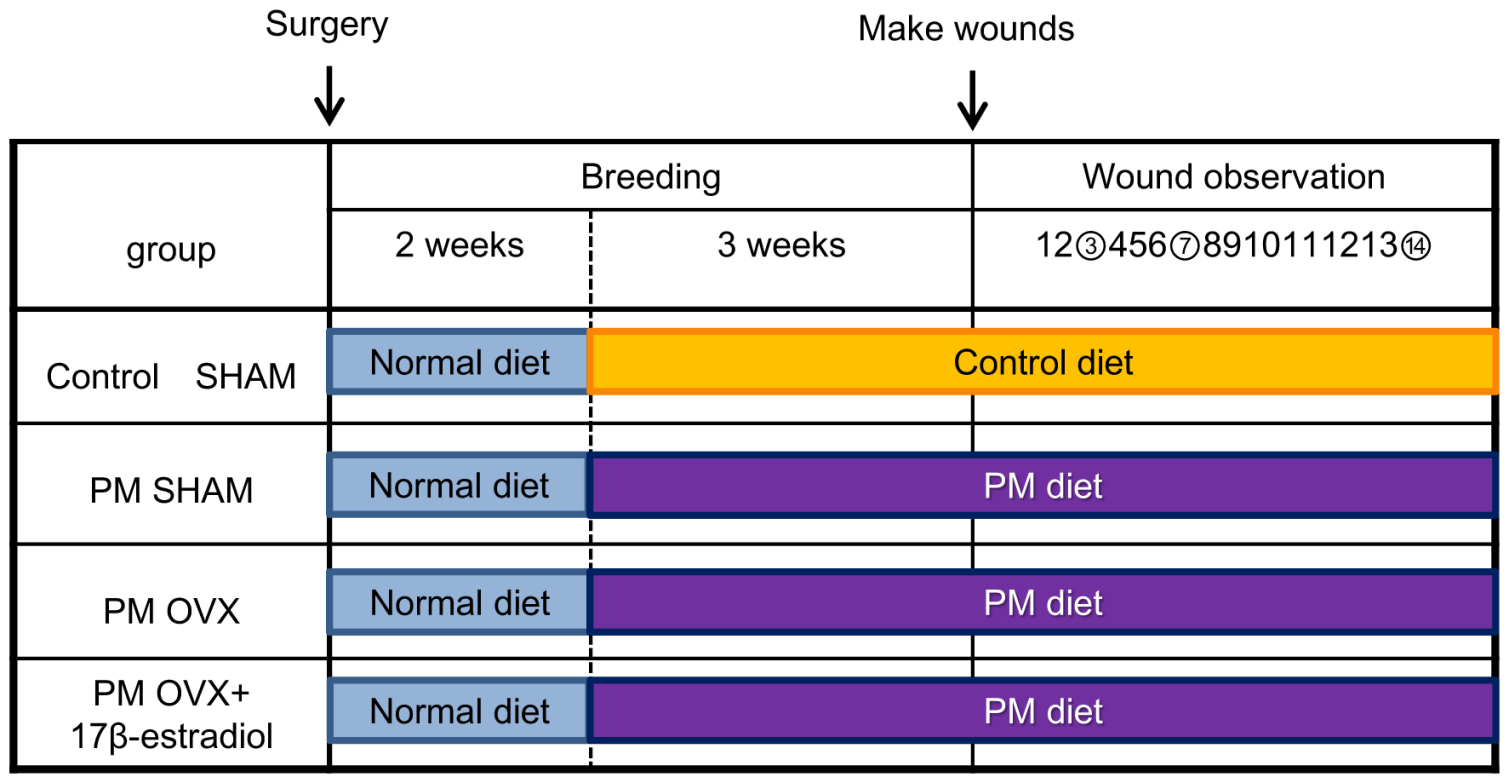
Research schedule. Mice were subjected to sham surgery (SHAM) or ovariectomy (OVX) and divided into four groups: Control SHAM group, protein malnutrition (PM) SHAM group, PM OVX group and PM OVX+17β-estradiol group. Three different colors show different diets. After surgery, all groups were fed a normal diet for 2 weeks (blue box) as a period of recovery from the surgery. Then, the Control SHAM group was fed on a control diet (orange box) for 3 weeks, but the other 3 groups were fed on a PM diet (purple box) for 3 weeks. At 5 weeks after surgery, two circular full-thickness skin wounds (4 mm in diameter) including the panniculus carnosus muscle on both sides of the dorsum of the mouse were made with a Kai sterile disposable biopsy punch. The process of wound healing was observed every day for 15 days, and wounds were harvested on days 3, 7 and 14 after wounding (circled numbers).

The mice were acclimated for 7 days before initiation of surgery. At 8 weeks old, the mice were anesthetized by intraperitoneal (IP) injection of pentobarbital sodium (0.05 mg/g weight) and the dorsum was shaved. Then, they were subjected to sham surgery (SHAM) or ovariectomy (OVX) according to OECD guidelines [27]. They were divided into four groups: Control SHAM group (n = 25), PM SHAM group (n = 24), PM OVX group (n = 20) and PM OVX+17β-estradiol group (n = 21). After surgery, all groups were fed a normal diet for 2 weeks as a period of recovery after the surgery. Then, the Control SHAM group was fed a control diet for 3 weeks, but the other 3 groups were fed a PM diet for 3 weeks, on the basis of previous research [9] (Fig. 1). Body weight and amount of diet intake were measured every week. Rodent diet consumption and protein consumption were calculated from the data for 3 weeks after feeding on Control diet or PM diet. The body weight variation was calculated using the initial (upon starting feeding on Control diet or PM diet) and final weights (day of wound preparation) of the animals in each group. At 13 weeks old (5 weeks after surgery), the mice (n = 10 in Control SHAM, 9 in PM SHAM and 6 in PM OVX and PM OVX+17β-estradiol groups) for assaying of total protein (TP) and albumin (Alb) were euthanized by a massive pentobarbital sodium IP injection. Plasma was prepared from each mouse's blood isolated through cardiac puncture and frozen until the time of assaying of TP and Alb. This was outsourced to the assay manufacturer (Mitsubishi Chemical Medience Co., Tokyo, Japan).

### Wounding

At 13 weeks old (5 weeks after surgery), under anesthesia with shaving, two circular full-thickness skin wounds (4 mm in diameter) including the panniculus carnosus muscle on both sides of the dorsum of the mouse were made with a Kai sterile disposable biopsy punch (Kai Industries Co. Ltd., Gifu, Japan). In the Control SHAM, PM SHAM and PM OVX groups, the wounds were covered with hydrocolloid dressing (Tegaderm; 3 M Health Care, Tokyo, Japan) to maintain a moist environment, and then the mouse was wrapped with sticky bandages (Meshpore Tape; Nichiban, Tokyo, Japan), which were changed every day. In the PM OVX+17β-estradiol group, wounds received the same treatment. However, after wound treatment, they were also treated with 0.01 g of 17β-estradiol gel (L'estrogel 0.06%; Bayer Yakuhin, Osaka, Japan). It was placed on clean gauze using a 1-mL syringe and applied to the skin on the back avoiding the wounds every day, and the gauze was covered with sticky bandages. The dose of 17β-estradiol was chosen with successful estrogen replacement confirmed by enzyme immunoassay on plasma samples and a gain of the uterine weight, as used in previous wound healing studies [23], [24], [28].

### Macroscopic observation

The day when wounds were made was designated as day 0, and the process of wound healing was observed from then until day 14 after wounding. Wound edges were traced on polypropylene sheets and photographs were taken every day. The traces on the sheets were captured with a scanner onto a personal computer using Adobe Photoshop Elements 7.0 (Adobe System Inc., Tokyo, Japan), and the areas of wounds were calculated using image analysis software Image J (National Institutes of Health, Bethesda, Maryland, USA). Wound area is shown as the ratio of wound area every day to the initial wound area on day 0 when the wound was made, according to our previous studies [23], [24], [28].

### Uterus assay

The mice were euthanized on days 3, 7 and 14 after wounding. The uterus was harvested according to OECD guidelines [27] and its wet weight was measured.

### Histological procedure and immunohistological staining

The mice were euthanized on days 3, 7 and 14 after wounding. The wound and the surrounding intact skin were harvested and each sample of wound and surrounding intact skin was bisected at the wound center. Each wound was stapled onto polypropylene sheets to prevent over-contraction of the sample and fixed in 4% paraformaldehyde for 17 hours. These samples were dehydrated in an alcohol series, cleaned in xylene and embedded in paraffin to prepare 5-µm serial paraffin sections. At least 6 serial sections near the center of the wound were obtained from one wound and stained according to the following methods. Sections of 5-µm thickness were subjected to hematoxylin and eosin (H&E) staining or Azan staining and immunohistologically stained with anti-neutrophil antibody at a concentration of 1∶100 (ab2557, Abcam Japan, Tokyo, Japan) for detecting neutrophils, anti-Mac-3 antibody at a concentration of 1∶100 (550292, BD Pharmingen, Tokyo, Japan) for detecting macrophages, anti-Ym1 antibody at a concentration of 1∶300 (#01404, StemCell Technologies, Tokyo, Japan) for detecting anti-inflammatory M2-like macrophages and anti-α smooth muscle actin (α-SMA) antibody at a concentration of 1∶300 (ab5694, Abcam Japan, Tokyo, Japan) for detecting myofibroblasts. Negative control slides were obtained by omitting each primary antibody.

### Microscopic observations

In normal cutaneous wound healing, the inflammatory phase is equivalent to day 3, the proliferation phase to day 7 and the remodeling phase to day 14. Therefore, we compared the four groups on days 3 and 7 in order to evaluate the inflammatory response and on days 7 and 14 to evaluate healing. Images were imported onto a computer using a digital microscopic camera (DP2-BSW Olympus, Japan). Measurements for the histological wound width and wound area were performed using DP2-BSW Olympus software with a ×4 objective upon HE staining: the histological wound width was determined by measuring the distance between wound edges and the histological wound area was calculated from wound edges below the necrotic tissue or new epithelium, extending to the level of the panniculus carnosus [29]. To analyze the numbers of neutrophils, macrophages and Ym1-positive cells in the wound area, each positive cell was counted using image analysis software Image J using a ×40 objective at five sites of the wound: two sites near the two wound edges and three sites around the center of the wound. The areas of these five sites were calculated on the monitor of the DP2-BSW and the total numbers of neutrophils, macrophages and Ym1-positive cells at the five sites were divided by the whole area of these five sites. Ym1-positive cell count/macrophage count (%) was calculated using the number of Ym1-positive cells per mm^2^ and the number of macrophages per mm^2^. To analyze the numbers of new blood vessels in granulation tissue, each new blood vessel was counted using image analysis software Image J with a ×40 objective upon HE staining at five sites of granulation tissue: two sites near the two wound edges and three sites around the center of the granulation tissue. The total number of new blood vessels at the five sites was divided by the total area of these five sites. Measurements for collagen fibers colored blue (collagen fiber pixels/total wound pixels) and myofibroblasts colored brown (myofibroblast pixels/total wound pixels) were performed using Adobe Photoshop Elements 7.0, according to our previous research [23], [24].

### Plasma TNF-α and CD163

The mice were euthanized on days 3 and 7 after wounding. Plasma was prepared from each mouse's blood isolated through cardiac puncture and frozen until the time of assays. Plasma TNF-α levels were determined by ELISA (R&D Systems, Tokyo, Japan) and plasma CD163 levels were also determined by ELISA (USCN Life Science Inc., Wuhan, China) according to the manufacturers' guidelines.

### Statistical analysis

Data are expressed as mean ±SD, analyzed using JMP 8.0.1 (SAS Institute Inc., Cary, NC, USA). Comparisons of means among multiple groups were performed with one-way ANOVA followed by post hoc pairwise comparisons using Tukey-Kramer multiple comparison test. And comparisons of means between day 3 and day 7 or day 7 and day 14 were performed with Student's t-test. A two-tailed p<0.05 was considered statistically significant.

## Results

### Body weight

**Figure 2:**
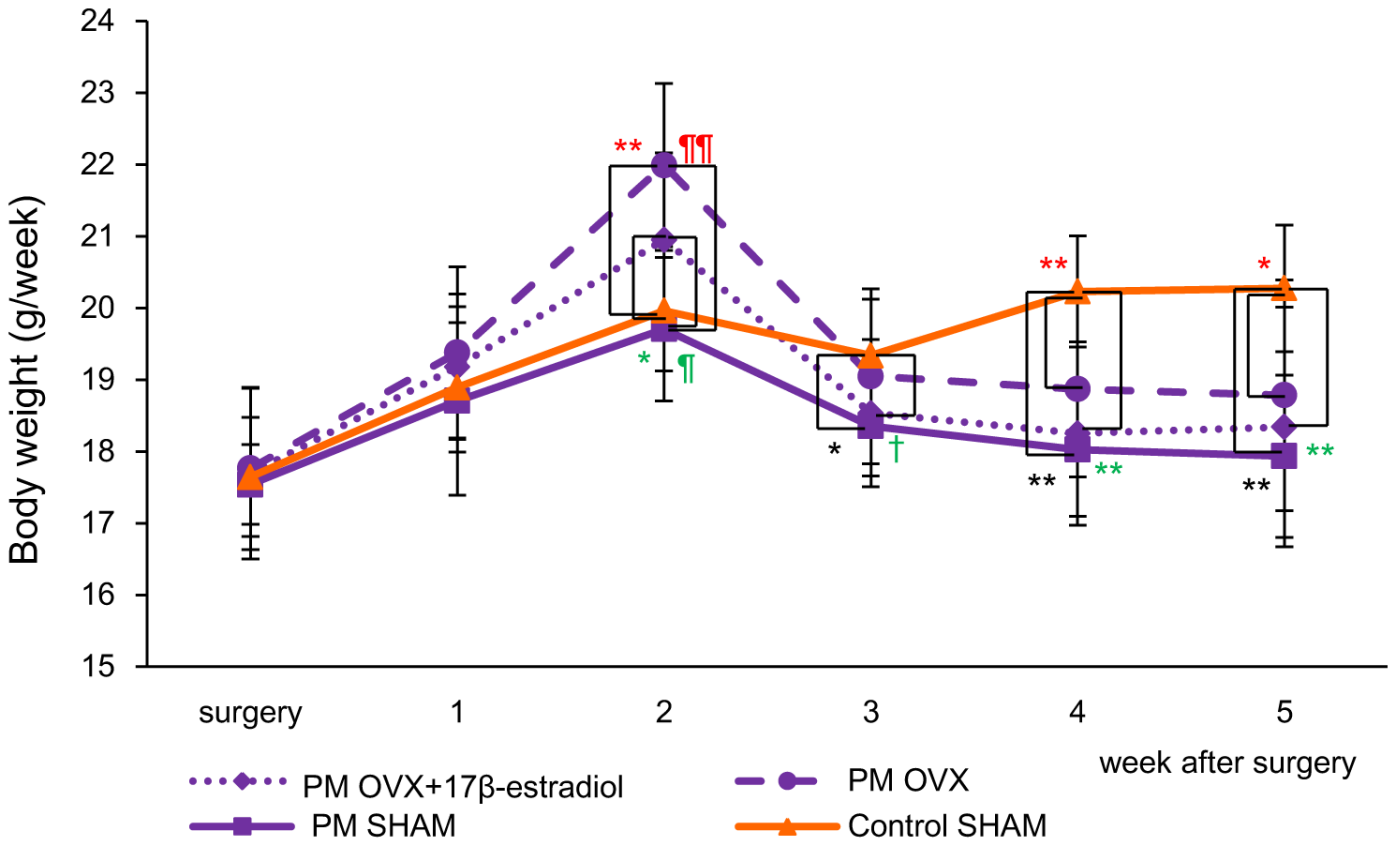
Body weight. The body weights of each group are shown on line graphs for each week. Values are expressed as mean ±SD, n = 15, in Control SHAM, PM SHAM and PM OVX+17β-estradiol groups (before treatment with 17β-estradiol) and 14 in the PM OVX group, ANOVA, Tukey-Kramer ^*^p<0.05, ^**^p<0.01 (in black): Control SHAM versus PM SHAM, ^*^p<0.05, ^**^p<0.01 (in red): Control SHAM versus PM OVX, ^†^p<0.1, ^*^p<0.05, ^**^p<0.01 (in green): Control SHAM versus PM OVX+17β-estradiol, ^¶¶^p<0.01 (in red): PM OVX versus PM SHAM and ^¶^p<0.05 (in green): PM OVX+17β-estradiol versus PM SHAM.

At the time of surgery, the body weight showed no significant differences among the four groups (17.6±0.83 g in the Control SHAM, 17.5±0.56 g in the PM SHAM, 17.8±1.13 g in the PM OVX and 17.7±1.19 g in the PM OVX+17β-estradiol groups). Then, for 2 weeks after surgery, upon feeding on a normal diet, the body weights in the PM OVX and PM OVX+17β-estradiol groups were significantly increased compared with those in the Control SHAM and PM SHAM groups (p<0.05). However, after feeding on Control diet (for Control SHAM) or PM diet (for PM SHAM, PM OVX and PM OVX+17β-estradiol groups), the mice fed a PM diet exhibited a drastic reduction in body weight on the day of wound preparation (body weight variation: −8.88±5.89% in the PM SHAM, −14.6±6.07% in the PM OVX and −13.0±6.33% in the PM OVX+17β-estradiol groups) (Table 3), and the body weights in the PM SHAM, PM OVX and PM OVX+17β-estradiol groups were significantly decreased compared with that in the Control SHAM group on the day of wound preparation (5 weeks after surgery) (p<0.05) (Fig. 2).

**Table 3:** Rodent diet consumption, protein consumption, body weight variation, TP and Alb among the four groups.

	Control	PM	PM	PM OVX+
	SHAM	SHAM	OVX	17β-estradiol
Rodent diet consumption (g/week/animal)	19.6±1.43	22.9±1.84	20.6±2.65	19.7±2.98
Protein consumption (g/week/animal)	3.92±0.28	0.69±0.06**	0.62±0.08**	0.59±0.09**
Body weight variation (%)	+2.72±1.87	−8.88±5.89**	−14.6±6.07**	−13.0±6.33**
TP (g/dL)	4.25±0.15	3.84±0.38*	3.80±0.28*	3.75±0.34*
Alb (g/dL)	3.46±0.13	3.08±0.31*	2.90±0.29**	2.92±0.29**

Data are expressed as mean ±SD. Rodent diet consumption and protein consumption were calculated from the data for 3 weeks after feeding on Control diet or PM diet. The body weight variation was calculated using the initial (upon starting feeding on Control diet or PM diet) and final weights (day of wound preparation) of the animals in each group. n = 15 in Control SHAM, PM SHAM and PM OVX+17β-estradiol groups and 14 in PM OVX group (rodent diet consumption, protein consumption and body weight variation) and n = 10 in Control SHAM, 9 in PM SHAM and 6 in PM OVX and PM OVX+17β-estradiol groups (TP and Alb). ANOVA, Tukey-Kramer.

*p<0.05,

**p<0.01versus Control SHAM.

### Food consumption, TP and Alb

Rodent diet consumption (g/week/animal) was 19.6±1.43 g in the Control SHAM, 22.9±1.84 g in the PM SHAM, 20.6±2.65 g in the PM OVX and 19.7±2.98 g in the PM OVX+17β-estradiol groups, with no significant differences among these four groups (Table 3). Protein consumption (g/week/animal) was 3.92±0.28 g in the Control SHAM, 0.69±0.06 g in the PM SHAM, 0.62±0.08 g in the PM OVX and 0.59±0.09 g in the PM OVX+17β-estradiol groups; that in the Control SHAM group was significantly higher than those in the other three groups (Table 3). Moreover, TP and Alb in the Control SHAM group were also significantly greater than those in the other three groups on the day of wound preparation (5 weeks after surgery) (p<0.05).

Malnutrition is defined as a state of deficient energy/protein intake or absorption, which is characterized by weight loss and changes in body composition [30]. Several previous studies compared body weight or TP and Alb to evaluate a protein malnutrition (PM) model, and reported that they were significantly decreased in mice fed a PM diet [8], [9], [31], [32]. Our results were the same. Therefore, it is indicated that our setting was appropriate and we could establish a protein malnutrition model.

### Uterine weight

We confirmed that the ovaries had been removed successfully in the PM OVX and PM OVX+17β-estradiol groups. The uterine weight in the PM OVX+17β-estradiol group was significantly greater than those in the other three groups (p<0.01) (Table 4). Moreover, the uterine weight in the Control SHAM group was also significantly greater than that in the PM OVX group (p<0.05) (Table 4). These results are almost the same as in our previous studies [23], [24], [28]. In addition, all of the gel that we applied on the skin on the back of the mouse was absorbed by the next day because the band covering the gel was not detached and the gel could not be felt. Therefore, it is thought that exogenous 17β-estradiol administration in OVX female mice led to its sufficient absorption.

**Table 4:** Uterine weight among the four groups.

	Control	PM	PM	PM OVX+
	SHAM	SHAM	OVX	17β-estradiol
Uterine weight (g)	0.044±0.008¶¶	0.038±0.009¶¶	0.022±0.006¶¶ *	0.100±0.038

Data are expressed as mean ±SD. n = 15 in Control SHAM, PM SHAM and PM OVX+17β-estradiol groups and 14 in PM OVX group. ANOVA, Tukey-Kramer.

*p<0.05: versus Control SHAM,

¶¶ p<0.01 versus PM OVX+17β-estradiol.

### Wound area

**Figure 3:**
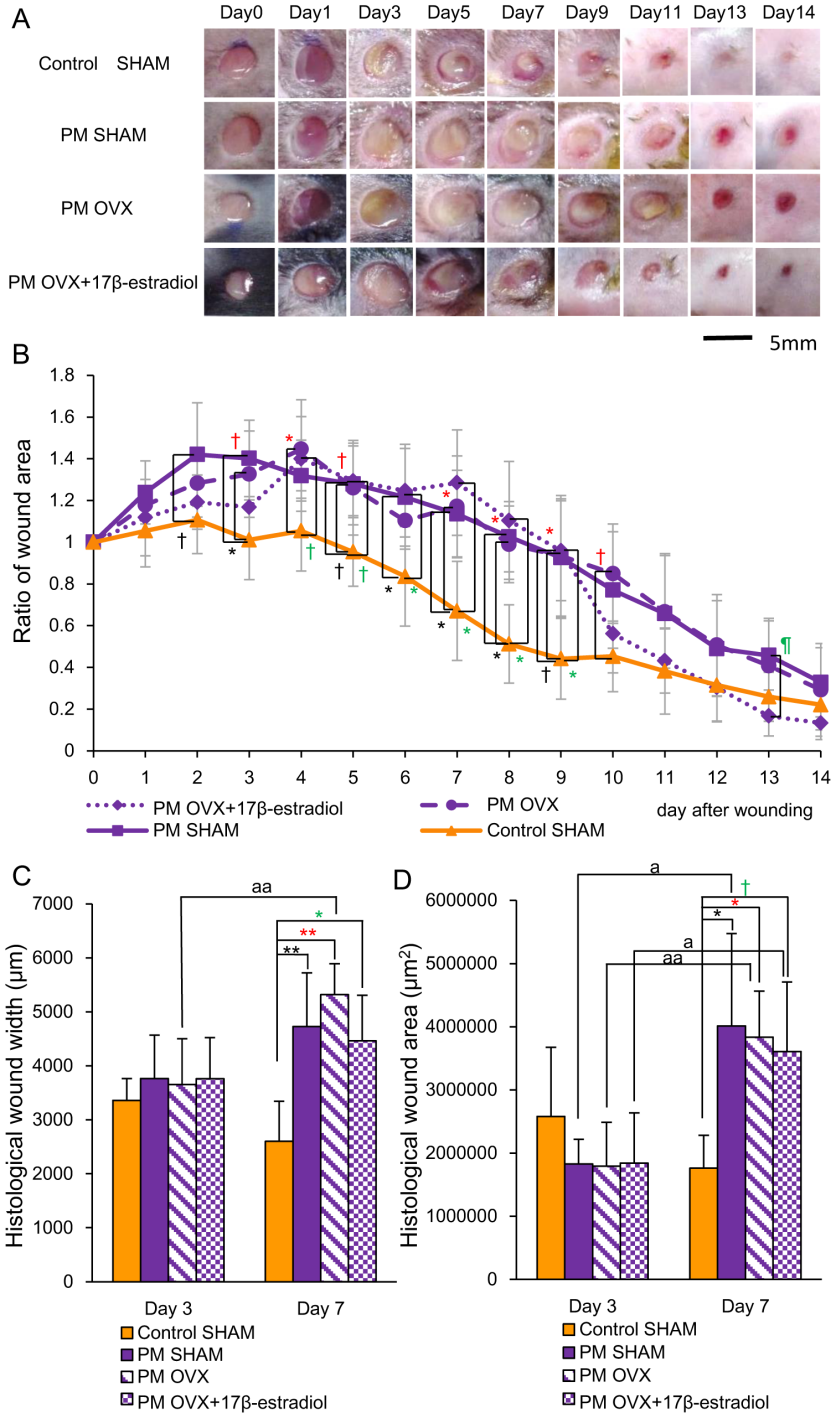
Macroscopic wound healing and histological wound width and wound area. (A) Four-mm-diameter wounds were inflicted and healing was recorded by photography. Bar, 5 mm. (B) Ratios of wound areas to initial area on day 0 are shown on line graphs for each day. (C) Histological wound width (µm) and (D) histological wound area (µm^2^) are shown on box graphs. Values are expressed as mean ±SD, n = 4–6 for each group, ANOVA, Tukey-Kramer ^†^p<0.1, ^*^p<0.05, ^**^p<0.01 (in black): Control SHAM versus PM SHAM, ^†^p<0.1, ^*^p<0.05, ^**^p<0.01 (in red): Control SHAM versus PM OVX, ^†^p<0.1,^*^p<0.05 (in green): Control SHAM versus PM OVX+17β-estradiol and ^¶^p<0.05 (in green): PM OVX+17β-estradiol versus PM SHAM, and t-test^ a^p<0.05, ^aa^p<0.01: day 3 versus day 7.

In the Control SHAM group, wound areas increased for 4 days and then decreased rapidly until day 9, after which they decreased slowly until day 14 and scars were formed. On the other hand, in the other three groups, wound areas increased for 8 days, and then those in the PM SHAM and PM OVX groups decreased gradually until day 14, but those in the PM OVX+17β-estradiol group decreased rapidly until day 13, after which they decreased slowly until day 14 and scars were formed (Fig. 3A and 3B). The ratio of wound area in the Control SHAM group was significantly smaller than that in the PM SHAM group on days 3 and 6–8 (p<0.05), and tended to be smaller than that on days 2, 5 and 9 (p<0.1). That in the Control SHAM group was also significantly smaller than that in the PM OVX group on days 4 and 7–9 (p<0.05), and tended to be smaller than that on days 3, 5 and 10 (p<0.1). Moreover, That in the Control SHAM group was significantly smaller than that in the PM OVX+17β-estradiol group on days 6–9 (p<0.05) and tended to be smaller than that on days 4 and 5 (p<0.1). There were no significant differences between the PM SHAM and PM OVX groups, or PM OVX and PM OVX+17β-estradiol groups throughout the entire period. On the other hand, the ratio of wound area in the PM OVX+17β-estradiol group was significantly smaller than that in the PM SHAM group on day 13 (p<0.05) (Fig. 3B).

Histological wound width in the Control SHAM group was significantly smaller than those in the PM SHAM (p<0.01), PM OVX (p<0.01) and PM OVX+17β-estradiol (p<0.05) groups on day 7 (Fig. 3C). On day 3, satisfactory establishment of granulation tissue had not occurred (data not shown). Histological wound area in the Control SHAM group was also significantly smaller than those in the PM SHAM and PM OVX groups (p<0.05) and tended to be smaller than that in the PM OVX+17β-estradiol group (p<0.1) on day 7 (Fig. 3D).

### Neutrophils, macrophages and Ym1-positive cell count/macrophage count (%)

**Figure 4:**
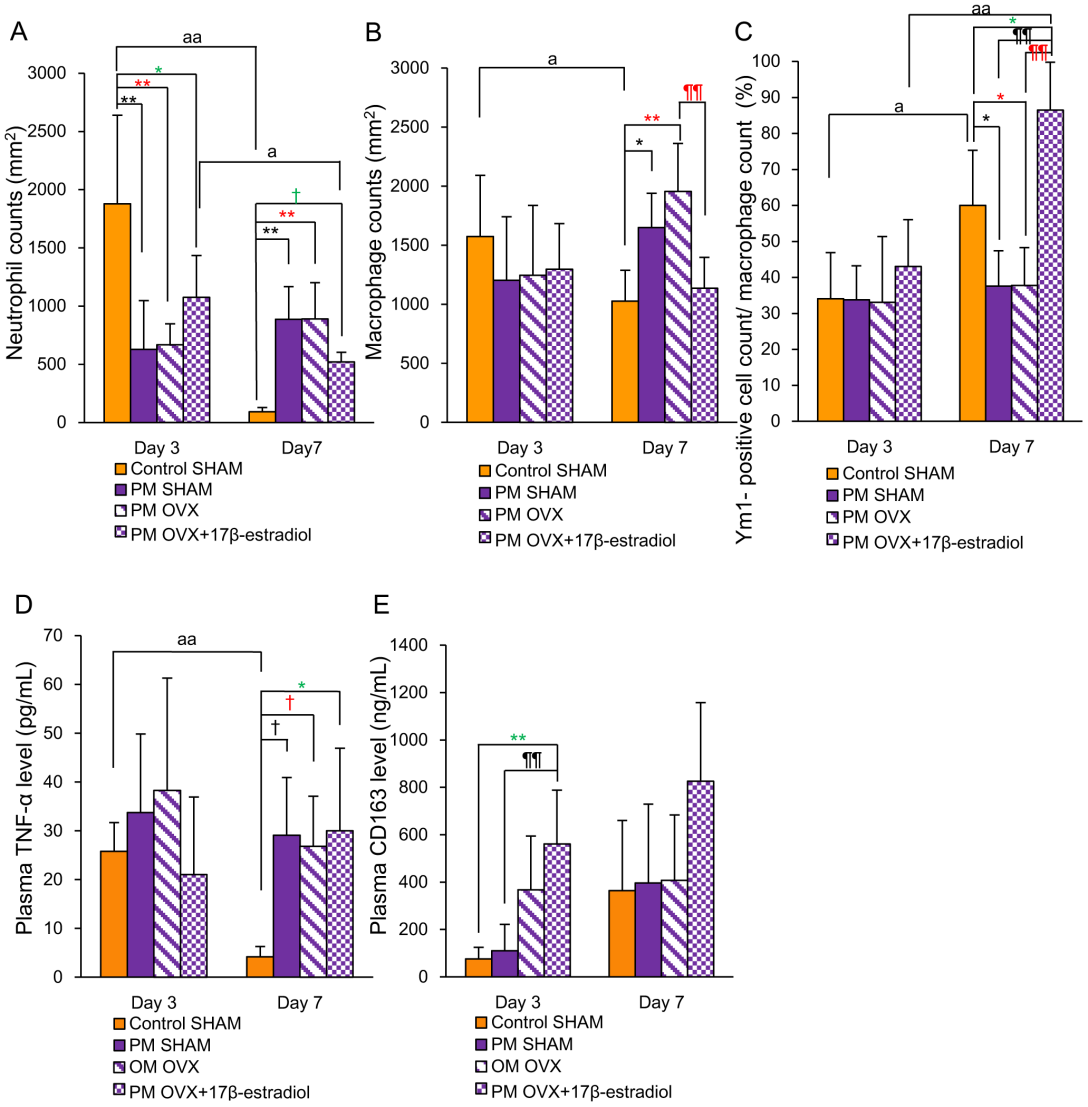
Neutrophils, macrophages, Ym1-positive cell count/macrophage count (%), plasma TNF-α and plasma CD163. (A) The number of neutrophils per mm^2^, (B) the number of macrophages per mm^2^, (C) Ym1-positive cell count/macrophage count (%), (D) systemic TNF-α level and (E) systemic CD163 level are shown on box graphs. Values are expressed as mean ±SD, n = 4–6 for each group, ANOVA, Tukey-Kramer ^†^p<0.1, ^*^p<0.05, ^**^p<0.01 (in black): Control SHAM versus PM SHAM, ^†^p<0.1, ^*^p<0.05, ^**^p<0.01 (in red): Control SHAM versus PM OVX, ^†^p<0.1, ^*^p<0.05 (in green): Control SHAM versus PM OVX+17β-estradiol, ^¶¶^p<0.01 (in black): PM OVX+17β-estradiol versus PM SHAM,^¶¶^p<0.01 (in red): PM OVX+17β-estradiol versus PM OVX, and t-test^ a^p<0.05, ^aa^p<0.01: day 3 versus day 7.

**Figure 5:**
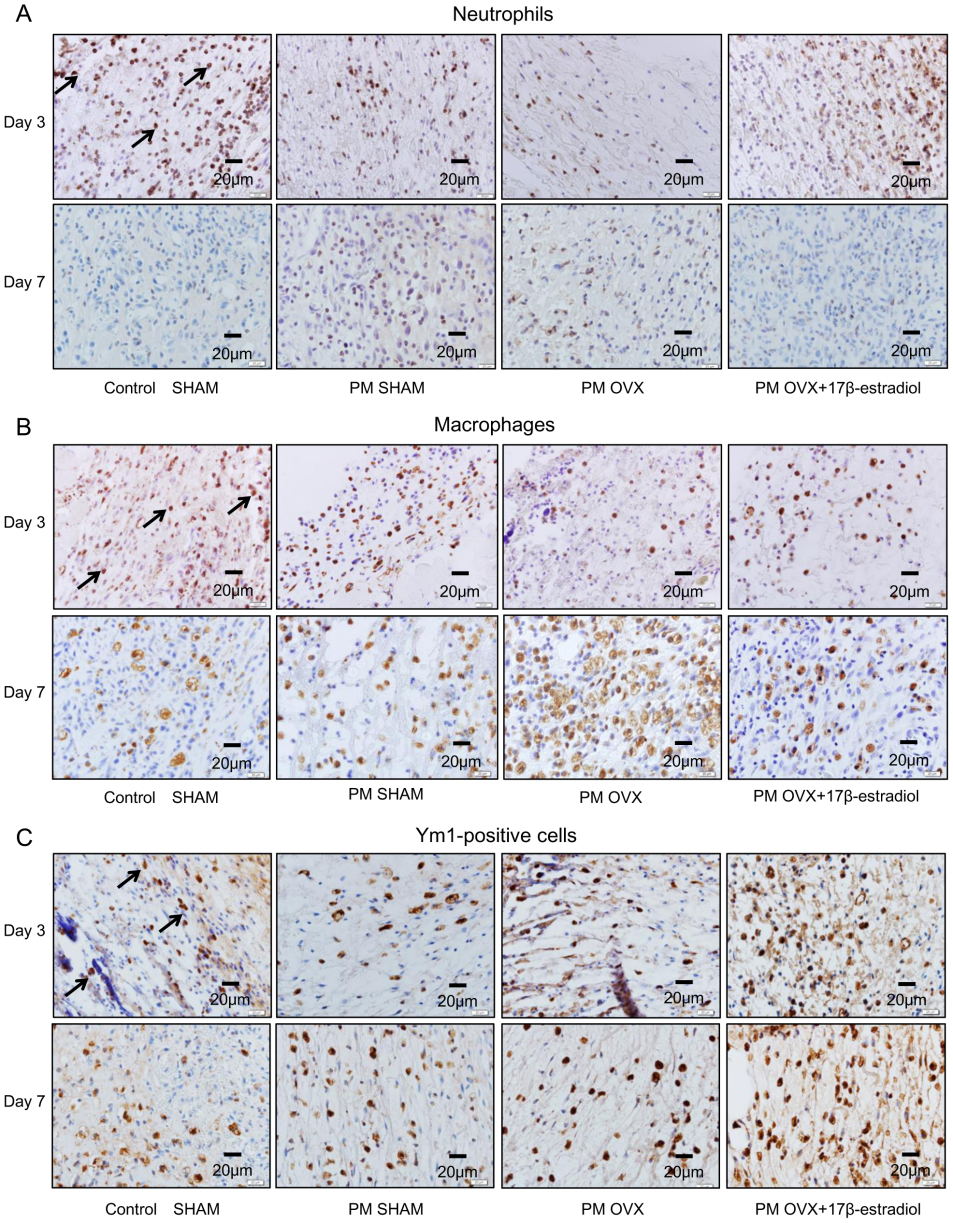
Immunohistological staining for neutrophils, macrophages and Ym1-positive cells on days 3 and 7. (A) Neutrophils (arrows) stained with anti-neutrophil antibody, (B) macrophages (arrows) stained with anti-Mac-3 antibody and (C) Ym1-positive cells (arrows) stained with anti-Ym1 antibody were observed in wound tissue on days 3 and 7.

The numbers of neutrophils in the Control SHAM (p<0.01) and PM OVX+17β-estradiol (p<0.05) groups were significantly decreased from days 3 to 7. In addition, that in the Control SHAM group was significantly larger than those in the PM SHAM and PM OVX groups on day 3 and significantly smaller than those on day 7 (p<0.01). In addition, that in the Control SHAM group was also significantly larger than that in the PM OVX+17β-estradiol group on day 3 (p<0.05), and tended to be smaller than that on day 7 (P<0.1) (Fig. 4A and 5A).

The number of macrophages in the Control SHAM group was significantly decreased from days 3 to 7 (p<0.05). In addition, there were no significant differences between Control SHAM and PM SHAM groups or Control SHAM and PM OVX groups on day 3, but this number in the Control SHAM group was significantly smaller than those in the PM SHAM (p<0.05) and PM OVX (p<0.01) groups on day 7. On the other hand, there were no significant differences in this variable between Control SHAM and PM OVX+17β-estradiol groups on days 3 and 7. Moreover, the number of macrophages in the PM OVX+17β-estradiol group was significantly smaller than that in the PM OVX group on day 7 (p<0.01) (Fig. 4B and 5B).

The Ym1-positive cell count/macrophage count (%) was significantly increased in the Control SHAM (p<0.05) and PM OVX+17β-estradiol (p<0.01) groups from days 3 to 7. This ratio in the Control SHAM group was significantly larger than those in the PM SHAM and PM OVX groups on day 7 (p<0.05). Moreover, this ratio in the PM OVX+17β-estradiol group was significantly larger than those in the PM SHAM and PM OVX groups (p<0.01) and the Control SHAM group (p<0.05) on day 7 (Fig. 4C and 5C).

### Plasma TNF-α and CD163 levels

Plasma TNF-α level in the Control SHAM group was significantly decreased from days 3 to 7 (p<0.01). It showed no significant differences among the four groups on day 3, but the level in the Control SHAM group was significantly lower than that in the PM OVX+17β-estradiol group (p<0.05), and tended to be lower than those in the PM SHAM and PM OVX groups (p<0.1) on day 7 (Fig. 4D). There were no significant differences among PM SHAM, PM OVX and PM OVX+17β-estradiol groups on day 7.

Plasma CD163 level in the PM OVX+17β-estradiol group was significantly higher than those in the Control SHAM and PM SHAM groups on day 3 (p<0.01). There were no significant differences among the four groups on day 7, but the value of CD163 in the PM OVX+17β-estradiol group was almost double those in the other three groups (Fig. 4E).

### Angiogenesis, collagen deposition and wound contraction

**Figure 6:**
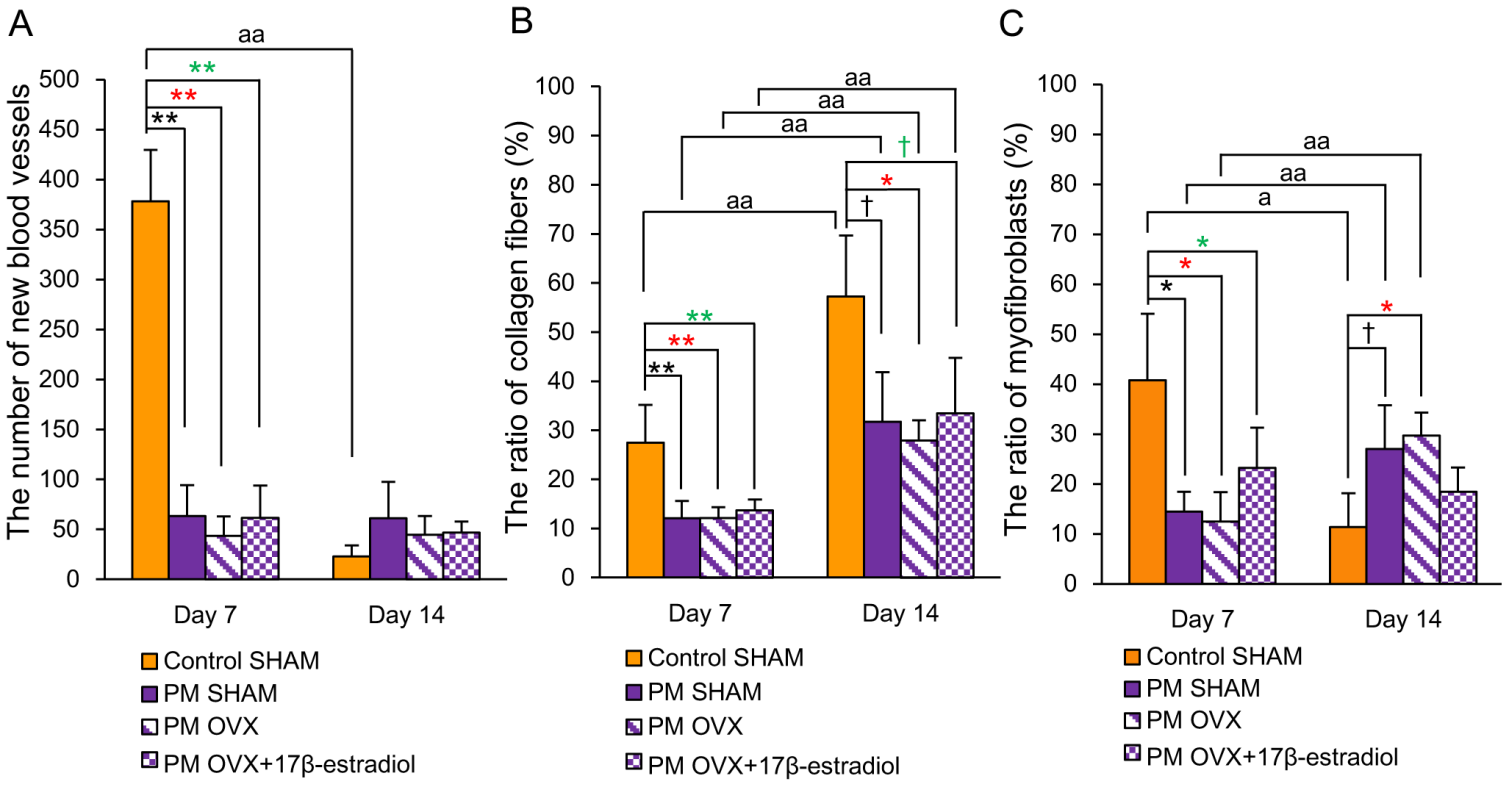
New blood vessels, collagen fibers and wound contraction. (A) The number of new blood vessels per mm^2^, (B) the ratio of collagen fibers (%) and (C) the ratio of myofibroblasts (%) are shown on box graphs. Values are expressed as mean ±SD, n = 3–5 for each group, ANOVA, Tukey-Kramer ^†^p<0.1, ^*^p<0.05, ^**^p<0.01 (in black): Control SHAM versus PM SHAM, ^*^p<0.05, ^**^p<0.01 (in red): Control SHAM versus PM OVX, ^†^p<0.1, ^*^p<0.05, ^**^p<0.01 (in green): Control SHAM versus PM OVX+17β-estradiol, and t-test^ a^p<0.05, ^aa^p<0.01: day 7 versus day 14.

**Figure 7:**
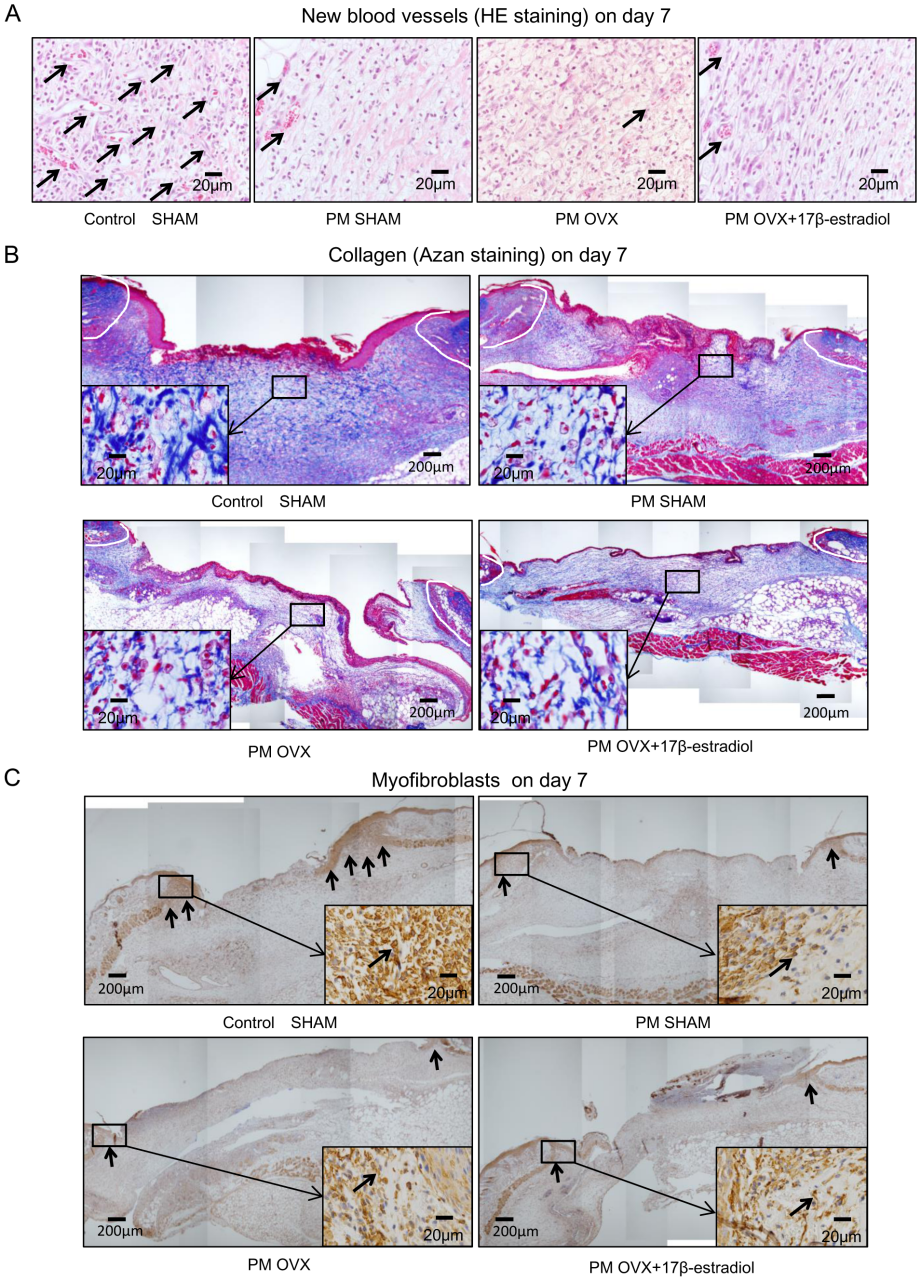
HE staining for new blood vessels, Azan staining for collagen fibers and immunohistological staining for myofibroblasts on day 7. (A) HE staining on day 7. Note that arrows indicate new blood vessels that show circular structures or include red blood cells. (B) Azan staining on day 7. Collagens stained blue were observed in the granulation tissue. Insets highlight regions of granulation tissue at higher magnification. (C) Myofibroblasts (arrows) stained with anti-α-SMA antibody on day 7. The myofibroblasts appeared at the wound edges on granulation tissue. Insets highlight regions of the wound edge on granulation tissue at higher magnification.

The number of new blood vessels in the Control SHAM group was significantly decreased from days 7 to 14 (p<0.01). That in the Control SHAM group was significantly larger than those in the other three groups on day 7 (p<0.01). On the other hand, there were no significant differences among PM SHAM, PM OVX and PM OVX+17β-estradiol groups on days 7 and 14 (Fig. 6A and 7A).

The ratios of collagen fibers were significantly increased from days 7 to 14 in the four groups (p<0.01). The ratio of collagen fibers in the Control SHAM group was significantly larger than those in the other three groups on day 7 (p<0.01), and significantly larger than that in the PM OVX group (p<0.05) and tended to be larger than those in the PM SHAM and PM OVX+17β-estradiol groups on day 14 (p<0.1). On the other hand, there were no significant differences in this variable among PM SHAM, PM OVX and PM OVX+17β-estradiol groups on days 7 and 14 (Fig. 6B and 7B).

The ratio of myofibroblasts in the Control SHAM group was significantly decreased from days 7 to 14 (p<0.05). On the other hand, those in the PM SHAM and PM OVX groups were significantly increased from days 7 to 14 (p<0.01), but that in the PM OVX+17β-estradiol group was decreased until day 14. The ratio of myofibroblasts in the Control SHAM group was significantly larger than those in the other three groups on day 7 (p<0.05). In addition, the ratio of myofibroblasts in the Control SHAM group was significantly smaller than that in the PM OVX group (p<0.05) and tended to be smaller than that in the PM SHAM group (p<0.1) on day 14, but it showed no significant difference between Control SHAM and PM OVX+17β-estradiol groups on day 14. On the other hand, there were no significant differences among PM SHAM, PM OVX and PM OVX+17β-estradiol groups on days 7 and 14 (Fig. 6C and 7C).

## Discussion

PM was previously shown to be characterized by prolonged and poor wound healing [33], [34]. Tsuda et al. reported that wound area was increased in mice fed a protein-free diet [8]. Lim et al. reported that wound size was increased and inflammatory response was delayed with decreased expression of TNF-α and neutrophil infiltration in mice fed a 5 g/kg protein diet in an early inflammatory phase [9]. Otranto et al. reported that inflammatory cells were present at a higher level and wound contraction, collagen deposition and neovascularization were impaired in a protein-restricted group [10]. Our findings, comparing Control SHAM and PM SHAM fed 30 g/kg PM diet groups, reflected these results. The ratio of wound area in the PM SHAM group was significantly larger than that in the Control SHAM group on days 3 and 6–8 in the inflammatory and proliferation phases. Histological wound width and wound area in the PM SHAM group were also significantly larger than those in the Control SHAM group on day 7. The numbers of neutrophils and macrophages and plasma TNF-α level in the Control SHAM group were significantly decreased from days 3 to 7, but those in the PM SHAM group were unchanged or increased. In addition, those in the PM SHAM group were significantly or tended to be larger than those in the Control SHAM group on day 7. Moreover, Ym1-positive cell count as an anti-inflammatory M2-like macrophage marker [19]–[22]/macrophage count in the Control SHAM group was significantly increased from days 3 to 7, but this ratio in the PM SHAM group was unchanged. Furthermore, this ratio in the Control SHAM group was significantly larger than that in the PM SHAM group on day 7. In addition, the number of new blood vessels and the ratio of myofibroblasts in the Control SHAM group were significantly larger than those in the PM SHAM group on day 7 and the ratio of collagen fibers in the Control SHAM group was significantly or tended to be larger than that in the PM SHAM group on days 7 and 14. These results show that cutaneous wound healing was delayed with protein malnutrition by increasing wound area, and delayed inflammatory response, angiogenesis, collagen deposition and wound contraction. Therefore, these results indicate the importance of protein for promoting cutaneous wound healing.

It was reported that cutaneous wound healing in young OVX female rodents under normal nutrition was delayed compared with that in SHAM mice by increasing inflammatory cells and the expression of TNF-α [12]–[18]. In addition, as mentioned above, cutaneous wound healing was delayed with protein malnutrition [8]–[10]. Therefore, it is thought that cutaneous wound healing in PM OVX mice was delayed compared with that in PM SHAM mice. However, no parameter for evaluating cutaneous wound healing, such as ratio of wound area, histological wound width and wound area, the numbers of neutrophils, macrophages and new blood vessels, Ym1-positive cell count/macrophage count, plasma TNF-α and CD163 levels, and the ratios of collagen fibers and myofibroblasts, showed a significant difference between the PM SHAM and PM OVX groups. Therefore, it is indicated that protein malnutrition had more influence than estrogen deprivation on cutaneous wound healing.

In normal nutrition, it has been reported that estrogen administration in OVX mice promoted cutaneous wound healing by reducing neutrophils, macrophages and the expression of TNF-α [13]–[18], and increasing Ym1-positive cells [16] and promoting collagen deposition [12], [14]. In addition, our previous research also reported that estrogen administration promoted cutaneous wound healing in OVX mice under normal nutrition by reducing neutrophils and macrophages [23], [24] and promoting collagen deposition [23], [24] and wound contraction [24] at 24 weeks and 40 weeks. Therefore, it is thought that the anti-inflammatory effect and cell proliferation action due to estrogen administration would be exhibited upon low-protein malnutrition, such as having a small amount of endogenous protein; cutaneous wound healing in the PM OVX+17β-estradiol group was promoted compared with that in PM OVX mice. However, the ratio of wound area, histological wound width and wound area, the number of new blood vessels and the ratios of collagen fibers and myofibroblasts showed no significant differences between the PM OVX and PM OVX+17β-estradiol groups. These findings indicate that estrogen administration alone could not accelerate the cutaneous wound healing like normal protein by improving the delay of wound healing in protein malnutrition. However, the following results from this research might show that estrogen administration minimizes the influence of protein malnutrition to induce normal wound healing: the number of neutrophils in the PM OVX+17β-estradiol group was significantly decreased from days 3 to 7 the same as that in the Control SHAM group; the number of macrophages in the PM OVX+17β-estradiol group was significantly smaller than that in the PM OVX group on day 7; Ym1-positive cell count/macrophage count in the PM OVX+17β-estradiol group was significantly increased from days 3 to 7 the same as that in the Control SHAM group; Ym1-positive cell count/macrophage count in the PM OVX+17β-estradiol group was significantly larger than that of not only the PM OVX group but also the Control SHAM group on day 7; plasma CD163 level as an anti-inflammatory M2-like macrophage marker [25], [26] in the PM OVX+17β-estradiol group was also higher than that in the Control SHAM group; and the ratio of myofibroblasts, which were shifted from fibroblasts by the effect of TGF-β1 [35], in the PM OVX+17β-estradiol group was decreased from days 7 to 14 (not significant), the same as that in the Control SHAM group, although that in the PM OVX group was significantly increased from days 7 to 14. Therefore, since anti-inflammatory M2-like macrophages that promoted tissue remodeling by producing anti-inflammatory cytokines and growth factors (TGF-β1, VEGF and PDGF) [21], [22] increased in the PM OVX+17β-estradiol group, wound areas in the PM OVX+17β-estradiol group were decreased rapidly from day 9 until day 13 and scars were formed like those in the Control SHAM group on day 14.

In summary, we found that protein malnutrition had more influence than estrogen deprivation on cutaneous wound healing. On the other hand, we also found that the appearance of anti-inflammatory M2-like macrophages was promoted by estrogen administration; however, it could not promote cutaneous wound healing upon feeding on the low-protein diet. Therefore, it may be confirmed that nutrition is more important for promoting cutaneous wound healing than estrogen administration.

However, cutaneous wound healing becomes disrupted and healing is delayed with advancing age [36]–[38]. Actually, more than 50% of the elderly in hospitals and institutions were found to be malnourished or at risk of malnutrition in developed countries [6], [7], and in Japan malnutrition was shown to be increased as the level of required care became high [7]. Therefore, we will conduct further research examining whether 17β-estradiol administration has effects to promote cutaneous wound healing in protein-malnourished older OVX female mice.
